# Using head-mounted eye tracking to examine visual and manual exploration during naturalistic toy play in children with and without autism spectrum disorder

**DOI:** 10.1038/s41598-021-81102-0

**Published:** 2021-02-11

**Authors:** Julia R. Yurkovic, Grace Lisandrelli, Rebecca C. Shaffer, Kelli C. Dominick, Ernest V. Pedapati, Craig A. Erickson, Daniel P. Kennedy, Chen Yu

**Affiliations:** 1 Department of Psychological and Brain Sciences, Indiana University, Bloomington, IN 47401 USA; 2 Department of Pediatrics, Cincinnati Children’s Hospital, Cincinnati, OH 45229 USA; 3 Department of Psychiatry and Behavioral Neuroscience, Cincinnati Children’s Hospital, Cincinnati, OH 45229 USA; 4 School of Medicine, University of Cincinnati, Cincinnati, OH 45229 USA; 5 Department of Psychological and Brain Sciences, University of Texas at Austin, Austin, Texas 78712 USA

**Keywords:** Psychology, Human behaviour

## Abstract

Multimodal exploration of objects during toy play is important for a child’s development and is suggested to be abnormal in children with autism spectrum disorder (ASD) due to either atypical attention or atypical action. However, little is known about how children with ASD coordinate their visual attention and manual actions during toy play. The current study aims to understand if and in what ways children with ASD generate exploratory behaviors to toys in natural, unconstrained contexts by utilizing head-mounted eye tracking to quantify moment-by-moment attention. We found no differences in how 24- to 48-mo children with and without ASD distribute their visual attention, generate manual action, or coordinate their visual and manual behaviors during toy play with a parent. Our findings suggest an intact ability and willingness of children with ASD to explore toys and suggest that context is important when studying child behavior.

## Introduction

Exploring the world through multiple sensory modalities, such as looking and touching, is crucial for children to learn about the world and the affordances it contains^1–3^. By attending to and acting upon the world, children create learning opportunities for themselves. Toy play is one everyday context in which young children learn about their worlds. Through both visual and manual exploration of objects during play, young children actively create rich sensorimotor experience that supports cognitive development^4^, including attention^5^, memory^6^, and perception^7^. Additionally, language learning in children is facilitated and fortified by exploration through play^8–11^. Finally, infant exploration elicits play- and language-promoting behaviors from the parent and these responsive parental behaviors in turn scaffold social development^12,13^. Taken together, multimodal exploration of objects through play supports cognitive, linguistic, and social development, and is therefore critical for typically developing (TD) children.

Given the importance of toy play for typical development, play has also been examined in children with autism spectrum disorder (ASD), a developmental disorder characterized by abnormalities in social communicative abilities and the presence of restricted and repetitive behavior and attention profiles. Research shows markedly lower levels of engagement and numbers of exploratory behaviors in toy play between children with ASD and TD children. Infants who later received an ASD diagnosis were found to orient to visual stimuli less frequently during play^14^. During exploration tasks, children with ASD looked at fewer objects^15^, explored fewer locations in a room^16,17^ and engaged in less exploratory behavior overall (quantified as total time spent looking around the room, manipulating objects, and walking toward containers in the room) compared to TD peers^17^. When playing with a parent for eight to 10 min, children with ASD played with fewer toys^18^ and spent less time playing with the toys compared to TD children^18,19^. Beyond the frequency of exploration, exploratory behaviors are also reportedly different between TD children and children with ASD. Visual exploration is atypical^20^, with children with ASD fixating on objects more frequently than their TD peers^14,21^. Exploration among children with ASD is often described as more unimodal (i.e. atypical visual fixation on an object)^22^, less varied, and more repetitive than that observed in TD children^18^. This research suggests that children with ASD may be less likely to engage in play and, when they do, are likely to explore objects in more restricted ways than TD children.

Atypical attention is one possible factor contributing to atypical play. An overall presence of atypical attention allocation has been observed in many screen-based eye-tracking studies (see a recent meta-analysis^23^). Specifically, attention allocation in individuals with ASD is narrower and less flexible^24^, with a tendency to explore fewer objects for longer durations and to engage in more individual looks to parts of these select objects^25^. A study of natural-scene viewing showed adults with ASD tended to explore less (as evidenced by a center bias), have longer saccades, fewer fixations, and longer fixation durations on objects^26^. However, other studies using arrays of objects found that young (2- to 5-yo) children with ASD do not look at fewer images overall in visual arrays but instead look at fewer images of low interest to individuals with ASD (e.g., food, clothes) and more images of high interest to individuals with ASD (e.g., trains, blocks)^27^. This may suggest that stimulus type and age matter when studying visual attention in individuals with ASD. Further, children and adults with ASD display difficulties with disengaging their attention from one stimulus to shift to another during screen-based eye tracking tasks^24,28–30^. Infants and children with ASD also display longer latencies to orient to visual stimuli^31,32^, suggesting that both disengaging and re-engaging with visual stimuli are impaired.

### Current study

**Figure 1:**
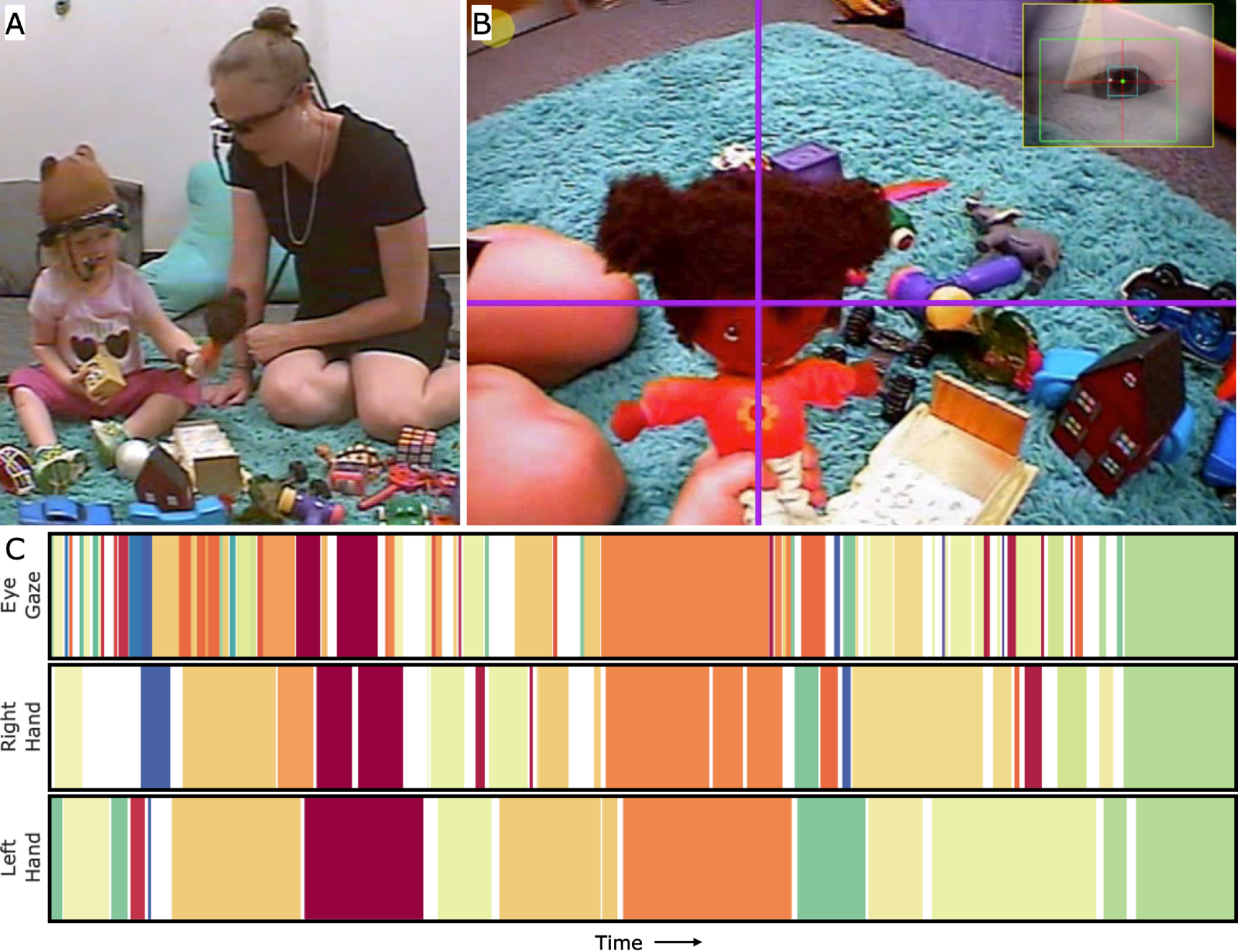
Experimental Set-Up and Data Processing. (**A**) Each dyad member wore a head-mounted eye tracker. Dyad members were seated next to each other and the toys were spread between them to play with following eye-tracker set-up. (**B**) The eye-tracker contains a small infrared camera that recorded each dyad member’s eye movements and a scene camera that recorded the visual scene in front of the participant. Gaze location is indicated with a purple cross-hair. (**C**) The child’s moment-to-moment gaze was manually coded by trained experimenters to indicate which toy they were looking at over the course of the play session. A time-series of visual fixation is shown, where each unique color represents one of the toys. Trained experimenters also indicated which toy was being touched by each hand over the course of the play session.

The overall aim of the present study is to examine whether and, if so, in what ways, children with ASD may generate different exploratory behaviors to toys in more natural, unconstrained contexts – the day-to-day contexts in which children use bodily actions to create their learning experience. To answer this question, the current study is the first study to use a novel experimental method wherein children with and without ASD are equipped with head-mounted eye trackers during naturalistic toy play. The head-mounted eye trackers capture not only the child’s view of their environment but also track where they look in the first-person view, moment by moment. Compared with screen-based eye tracking, head-mounted eye tracking records gaze data with a similar high resolution but allows the children to actively move their bodies and play with toys through their manual actions (See Fig. 1).

Most of the eye tracking studies examining children with ASD are screen-based, which is different than toy play in several critical ways:

1. **Space**: Screen-viewing experiments are carefully designed so that the background of the display is clean and that targets of interest are clearly separated in space. In everyday toy play, the environment is usually cluttered with multiple toys in close proximity to each other in space (i.e., when children stack one toy on the top of the other). Further, children participating in everyday toy play are able to move around in and explore their environment in a way that is not allowed in screen-based experiments.
2. **Time**: Screen-based experiments are usually composed of discrete trials in which certain stimuli are repeatedly displayed to elicit children’s attention; in contrast, toy play in naturalistic contexts is free-flowing as children are continuously acting upon and perceiving objects. Children are therefore able to select stimuli that are interesting to them at any given time throughout the experiment.
3. **Task**: Attention tasks in screen viewing are often designed to be simple to ensure that participants focus on the designated task; in contrast, children in toy play have their own goals and dynamically adjust their attention and actions to pursue these goals.

The differences described above raise two crucial questions that we aimed to answer in the present study: 1) How do children with ASD distribute their visual attention, generate manual action, and coordinate their visual and manual behaviors during naturalistic toy play in a naturalistic environment? and 2) Can the previous findings of atypical attention observed from screen-based laboratory experiments be generalized to more naturalistic everyday contexts, such as toy play? To answer these questions, we first investigated how children with and without ASD distribute their visual attention over a set of toys. If attention allocation during play is different between the TD and ASD groups, it would suggest that patterns of atypical attention allocation observed during screen-based eye tracking studies may be the underlying mechanism that gives rise to atypical play and exploration in children with ASD. If attention allocation is not different between the two groups, it would suggest that the ability to act on and explore toys may drive visual attention, and further suggest that atypical exploration may not emerge solely from visual attention patterns. To further understand sensorimotor behaviors in these naturalistic contexts, we next investigated the role of manual action in driving visual attention. If there are differences between the two groups in the coordination of visual attention with manual action, it would suggest that sensorimotor dynamics may lie at the core of atypical exploratory behavior in ASD. If the two groups have similar visual-manual coordination, it would suggest an ability and willingness for children with ASD to engage in typical object exploration.

## Results

### Visual attention

#### Analysis overview

The goals of the current study were to investigate visual attention, manual action, and visual-manual multimodal (hand-eye) coordination during a short period of naturalistic toy play in 14 ASD dyads and 15 TD dyads. In this section, we examine the ways that children with and without ASD visually explore a naturalistic play environment. We first investigated base rates of visual attention during the play session—how much time was spent looking at toys and how many unique toys were viewed. Similar amounts of time spent looking at toys, however, could be the result of very different visual exploration patterns (few long looks versus many short looks). Therefore, we next investigated how many looks were generated per minute and the median look duration. We then investigated different visual exploration patterns by comparing the distribution of looks to toys over time across the two groups. Finally, we investigated if there were differences between groups in a preference for “few” versus “many” toys by looking at the distribution of visual attention over toys.

#### Proportion of time, number and duration of looks to toys during play

**Figure 2:**
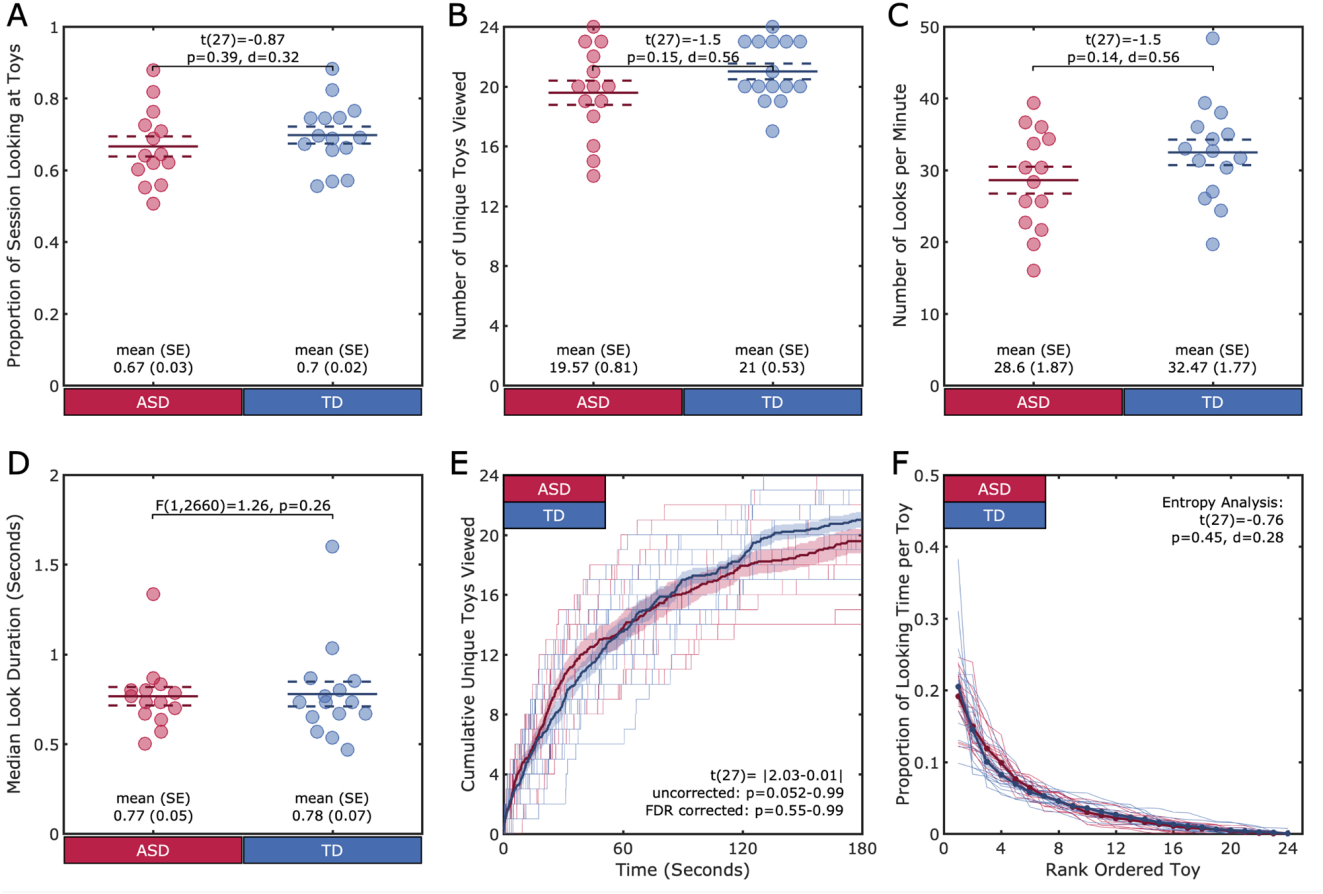
Visual Attention. (A-D) Each individual is represented by a circle. The group mean is represented by the solid line, and the standard error is represented by the dotted lines. (A). Groups did not differ on the proportion of the play session that each child spent looking at toys. (B) Groups did not differ on the number of unique toys (in the set of 24) that each child looked at during play. (C) Groups did not differ on the number of looks to toys that they generated per minute, (D) nor on the median duration of looks to toys. (E-F) Each individual is represented by a thin line. The bold lines represent the group means, and the shaded region represents the standard error. (E) The x-axis is the session time in seconds, and the y-axis is the cumulative unique number of toys viewed, such that each participant’s line increases by 1 each time they view a toy that they have not yet viewed. There were no differences between groups in the rate of looking at previously unviewed toys. (F). The x-axis is the rank ordered toy, such that for each child their most looked at toy is ranked 1 and their least looked at toy is ranked as 24. The y-axis is the proportion of the session spent looking at that toy. There were no differences between groups in the amount of the session spent looking at any of the toys, in rank order.

We first determined the proportion of the session that each child looked at the toys as opposed to looking at the parent’s face or non-target areas (the room, the wall, the rug, etc.). Children with ASD, on average, spent 67% of time (SD ±11%) looking at the toys during a 3-min play session. There was no difference in the amount of time spent looking at toys between children with ASD and TD children (TD 70% (8%); t(27) = -0.87, p = 0.39, d = 0.32) (See Fig. 2A). Within the first 3 min of the play session, both groups looked at a similar number of toys (ASD: 19.57 toys (3.03); TD: 21.00 (2.05); t(27) = − 1.5, p = 0.15, d = 0.56). (See Fig. 2B). Children in both groups visually explored more than 20 unique toys out of 24 toys available.

Next, we examined whether the similarities between groups in the number of toys viewed was driven by similar durations and numbers of looks to toys. Children with ASD produced 28.60 looks per minute (SD ±7.00) which is comparable to the number of looks produced by TD children (TD: 32.47 (6.86); t(27) = − 1.50, p = 0.14, d = 0.56) (See Fig. 2C). We next ran a linear mixed effects (LME) model on look duration with fixed effects for group and random effects for participant to account for the variability in look duration for each participant. An ANOVA on the LME model revealed no differences between groups, F(1,2660) = 1.26, p = 0.26 (See Fig. 2D). Children in both groups generated many short looks by switching between toys throughout the play session and, therefore, displayed similar patterns of visual exploration.

#### Distribution of looks over time

To examine not just the number of unique toys viewed, but the rate at which children in both groups explored new toys, we created cumulative distribution functions for each individual child. For each participant, we quantified how many unique toys they had viewed as a function of time (1 second bins) across the interaction. As shown in Fig. 2E, children in both groups visually explored more than 12 unique toys (over half of the available toys) within the first minute of play. As the play proceeded, the rate of exploring new toys decreased. Nonetheless, they attended to approximately 20 unique toys at the end of a play session. T-tests were conducted at each second of the data stream (181 total tests) to assess differences in the rate of exploration from second to second. Results indicated that there were no differences between groups in the number of toys that children had seen at any time point (t(27) = |2.03-0.01|, uncorrected p = 0.05-0.99; when p values were adjusted using a step-wise false discovery rate (FDR) procedure^33^, p = 0.55-0.99). To ensure that the rate of looking at toys was not driven by an individual toy that deferentially attracted gaze across groups, we compared the order (i.e., viewed first, second, third) that each child looked at each toy across groups. No toys were consistently looked at earlier in the session for one group compared to the other (t(27) = |1.52-0.02|, uncorrected p = 0.14-0.98; FDR corrected all p = 0.98). Thus, children within each group and between the groups showed similar rates of toy exploration over time. Moreover, there is no group difference between the children with ASD and TD children in terms of how they visually explored new objects throughout the play session.

#### Distribution of looks over toys

We next examined whether attention was allocated to the toys with equal frequency and whether there were any differences between children with and without ASD in the distributional properties of visual attention over the toys. To assess this, we measured proportion of looking time spent on each of the unique toys viewed. For each participant, the toys that they looked at were rank ordered from most to least attended. As shown in Fig. 2F, children in both groups spend over 50% of their looking time attending to four toys, and the remaining time looking at other toys. An entropy analysis was conducted on each individual’s gaze distribution to understand the degree of randomness of their attention to toys. A low entropy value would signify high selectivity - the majority of this child’s attention would be spent on one or two toys, with very little time spent attending to the other toys. A high entropy value would signify that the child’s attention would be spread evenly among the toys they viewed, and the probability that the child was playing with any one particular toy at any moment would be low. The maximum entropy for looks distributed over 24 toys is 3.18 if a child spent the same amount of time evenly on each of the 24 toys.

Children with ASD had a mean entropy value of 2.50 (SD ±0.22), suggesting a general preference for about four toys, with lower levels of looking to the other toys. There was no difference in entropy between groups (TD: 2.56 (0.19); t(27) = -0.76, p = 0.45, d = 0.28). Further, no particular toys were consistently looked at more for one group compared to the other (t(27) = |1.53-0|, uncorrected p = 0.14-0.99; FDR corrected all p = 0.99).

In summary, based on multiple measures derived from gaze data, we found that children with ASD distributed their visual attention in strikingly similar ways to TD children. They rapidly switched their attention among toys and generated more than 20 looks per minute. With 24 available toys, they explored the same number of toys at a similar rate over the entire session as their TD peers did. Finally, they didn’t distribute their attention evenly over the attended objects, and instead selectively attended to a few select objects much more than others which was similar to the TD group.

### Manual action

During free-flowing toy play, children not only look at toys but also actively generate actions on those toys (i.e., reaching for them, holding them, moving them from one location to another, or stacking them). A growing body of research using head-mounted cameras and head-mounted eye trackers shows that one toy typically dominates the child’s view during play at any one time because the child’s manual actions on the toy bring it closer to the child’s eyes and body, essentially reducing visual clutter^11^. Thus, manual activity can be viewed as a part of the attention system because it plays a role in selecting an object of interest and filtering out other objects in view^34^. This section reports a set of results on manual activities of toys by following the same structure used in reporting visual attention in the previous section. Similar to the measures reported for visual attention analyses, we calculated overall proportion of time, duration and frequency of manual actions, as well as the base rates and distributions of manual activities over time and over toys. We focused on overall manual activities by annotating when the child’s hands made contact with a toy. Since this annotation scheme means to include all kinds of manual actions that occurred in free toy play, including object touches, object holds and object manipulations, we will use the term “in-hand” to refer to any manual actions, including touching, holding, and manipulating objects.

#### Proportion of time, number and duration of manual actions on toys during play

**Figure 3:**
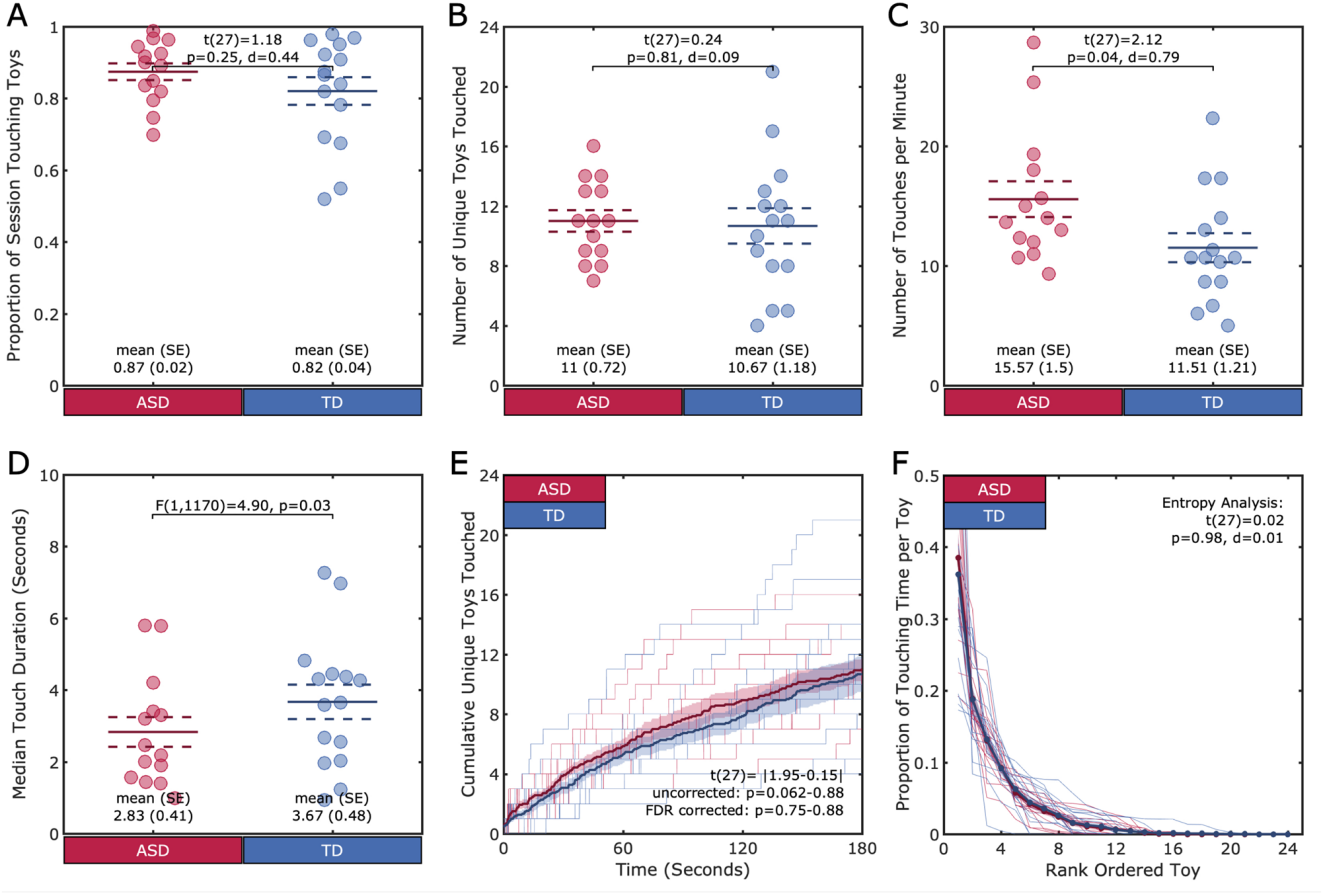
Manual Action. (**A**–**D**) Each individual is represented by a circle. The group mean is represented by the solid line, and the standard error is represented by the dotted lines. (**A**). Groups did not differ on the proportion of the play session that each child spent manually acting on toys. (**B**) Groups did not differ on the number of unique toys (in the set of 24) that each child manually acted upon. (**C**) Children with ASD generated more manual actions on toys per minute compared to their TD peers and (**D**) they generated shorter actions. (**E**–**F**) Each individual is represented by a thin line. The bold lines represent the group means, and the shaded region represents the standard error. (**E**) The x-axis is the session time in seconds, and the y-axis is the cumulative unique number of manually acted upon toys. There were no differences between groups in the rate of manually acting on previously un-acted on toys. (**F**). The x-axis is the rank ordered toy and the y-axis is the proportion of the session spent manually acting on that toy. There were no differences between groups in the amount of the session spent manually acting on any of the toys, in rank order.

We first calculated the proportion of time that each child spent acting on the toys. Children with ASD spent an average of 87% (SD ±7%) of the session actively using their hands to generate manual actions on toys. There was no difference in the amount of time that children with ASD and TD children spent manually acting on toys during the session (TD 82% (15%); t(27) = 1.18, p = 0.25, d = 0.44) (See Fig. 3A), suggesting that children with ASD are as manually active as TD children. We next determined how many unique toys were acted upon by each participant during the play session. Children with ASD acted on 11 toys (SD ±2.69) on average during the 3 min play session, which was comparable to TD children (TD: 10.67 (4.57); t(27) = 0.24, p = 0.81, d = 0.09) (See Fig. 3B). Children in both groups explored fewer objects through manual exploration than through visual exploration (ASD t(13) = − 10.95, p<0.001, d = 2.99; TD t(14) = − 8.49, p<0.001, d = 2.92).

With the same total amount of manual activities, we next analyzed the frequency and duration of manual actions on toys to test the possibilities that children could generate few actions with long durations or more actions with short durations. Children with ASD generated 15.57 (SD ±5.61) individual actions to toys per minute and the median duration of each action was 2.83s (SD ±1.53). Thus, their manual actions lasted much longer than their looks (ASD t(13) = 5.25, p<0.001, d = 1.87; TD t(14) = 5.73, p<0.001, d = 2.18). We found a significant difference between groups in the number of toys touched per minute (TD: 11.51 (4.69); t(27) = 2.12, p = 0.04, d = 0.79) (See Fig. 3C–D). We next ran a linear mixed effects model on touch duration with fixed effects for group and random effects for participant. An ANOVA on the LME revealed a difference between groups, F(1,1170) = 4.90, p = 0.03. Children with ASD generate more, shorter manual actions on toys relative to TD children.

#### Distribution of manual activities over time

We next examined the rate at which children explored unique toys during the play session. To do this, we calculated a cumulative distribution function for each child in 1 second increments, with the number of unique in-hand toys as a function of time elapsed during the session (See Fig. 3E). Children with ASD generated manual actions toward 5 objects within the first 30 seconds in a play session, and 5 more unique toys by the end of the first 3 min. Compared with the cumulative distribution on visual exploration shown in Fig. 2E, the distribution of manual action (shown Fig. 3E) is less skewed, suggesting that children manually explored a smaller number of toys compared with their visual exploration.

T-tests were conducted at each second of the data stream (181 total tests). A comparison of children with ASD and with TD showed no differences between the two groups in the number of in-hand toys that children manually explored at any time point (t(27) = |1.95-0.15|, uncorrected p = 0.06-0.88; FDR corrected p = 0.75-0.88). To ensure that the rate of in-hand toys was not driven by any individual toy, we compared the order (i.e., touched first, second, third) that each child touched each toy across groups. No toys were consistently manually acted upon earlier in the session for one group compared to the other (t(27) = |2.78-0.04|, uncorrected p = 0.02-0.97; FDR corrected p = 0.43-0.97). This result suggests a lack of a systematic group difference in the way that the children with ASD and TD children distributed their manual actions to objects.

#### Distribution of manual activities over toys

To determine the proportion of time that each child spent manually acting on each toy, we rank ordered the in-hand objects for each child by proportion of the total touching time. As shown in Fig. 3F, children in both groups spend over 50% of their manual action time acting on two toys, and the remaining time acting on other toys. We next calculated the entropy over the in-hand object distribution. As before, a high entropy value suggests an equal distribution of action across all toys, whereas a low entropy value suggests a more unequal distribution of action. The maximum entropy for touching of 24 toys is 3.18. The distribution of touches to toys for the children with ASD had an average entropy of 1.80 (SD ±0.39), suggesting that their actions on toys was skewed towards more time spent acting on very few toys. There was no difference in entropy between groups (TD: 1.79 (0.58); t(27) = 0.02, p = 0.98, d = 0.01) suggesting that both ASD and TD children distribute their actions to toys similarly. Further, no toys were consistently acted on more in one group compared to the other (t(27) = |2.59-0|, uncorrected p = 0.03–1; FDR corrected p = 0.60–1). Children with ASD and TD children distribute their manual action across many toys, but both groups selected 1 or 2 preferred toys, reflecting a similar pattern of exploration. Compared to the entropy of children looking to toys, entropy of children acting on toys is more selective.

We saw that children with ASD generated more, shorter manual actions to toys compared to their TD peers. However, there were no differences in manual exploration between the two groups. With 24 available toys, children with ASD and their TD peers explored similar numbers of toys at a similar rate over the session as their TD peers. Further, they selectively acted on some toys much more than others. We saw an overall lower rate of manual exploration than visual exploration across groups, reflecting that manual actions unfold at a slower timescale than visual attention.

### Visual-manual multimodal exploration

Recent studies show that children’s manual actions to objects during toy play create many different views of the same object, which facilitates both visual object recognition and early word learning^35–37^. In light of those findings, we next examined how children with ASD coordinate their eye and hand movements during object exploration. The results from the previous sections show that visual and manual exploration seem to be unimpaired in children with ASD during naturalistic toy play when assessed independently. Nonetheless, it is an open question whether the coordination of visual and manual exploration (hand-eye coordination) is different between groups. To answer this question, we analyzed the distribution of their visual attention when they are manually acting on toys, as well as the dynamics of looking to in-hand objects before, during and after manual actions.

#### Proportion of time in hand-eye coordination

**Figure 4:**
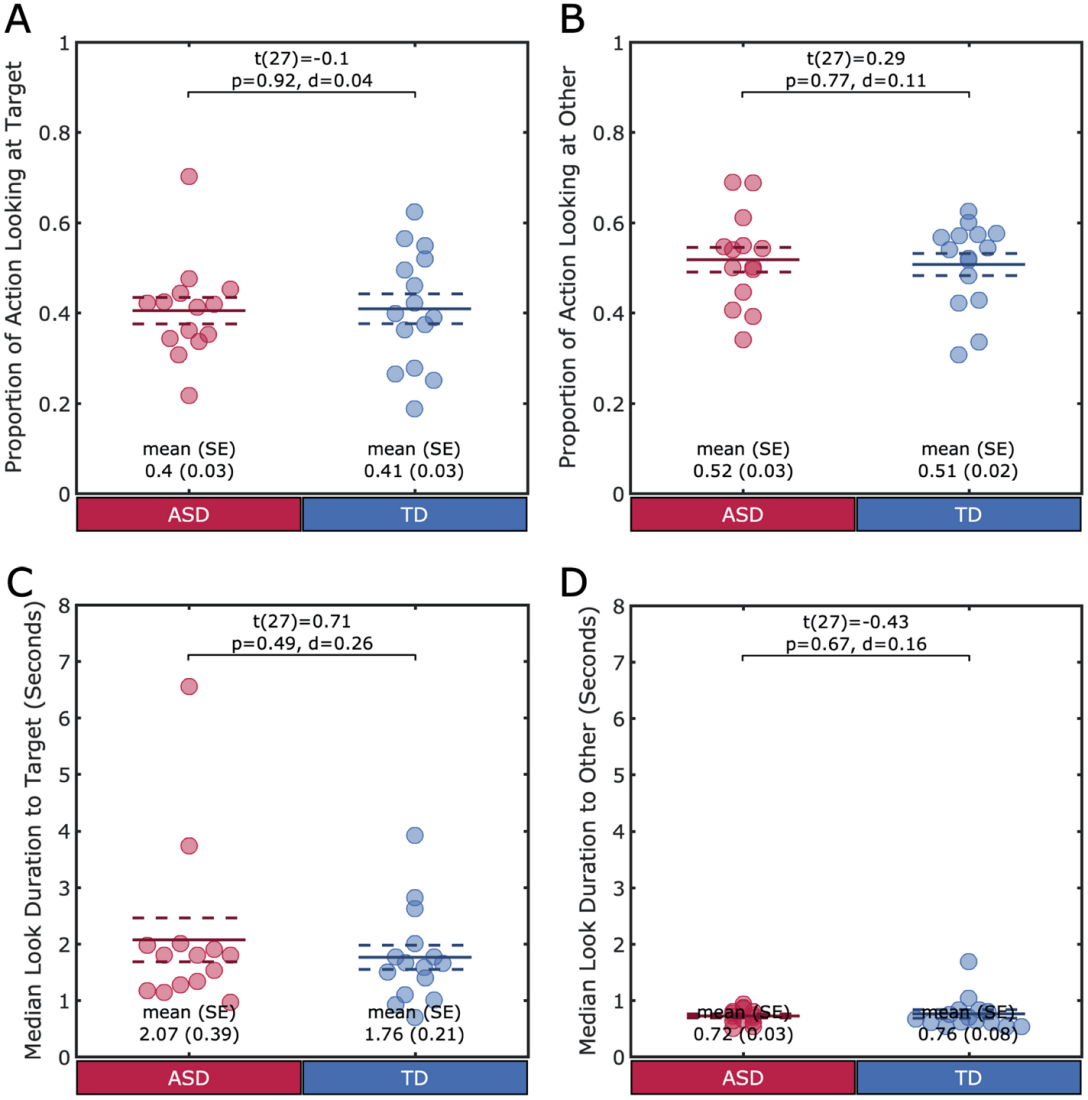
Hand-Eye Coordination. (**A**–**D**) Each individual is represented by a circle. The group mean is represented by the solid line, and the standard error is represented by the dotted lines. (**A**) Groups did not differ in the proportion of time acting upon a toy that they spent looking to the target toy (**B**) or to another toy. (**C**) Groups also did not differ in the duration of looks to the acted upon toy (D) or to the duration of looks to a non-in-hand toy while acting on a different toy.

We first aimed to understand how frequently children are visually engaging with a toy that is in-hand (i.e., being touched or manipulated in some way) as opposed to visually exploring toys that are not in-hand. We measured the proportion of time where a child had at least one toy in-hand and was looking to at least one of the in-hand (“target”) toys or looking to non-manipulated (“other”) toys. Children with ASD looked at target toys for 40% (SD ±11%) of the time a toy was in-hand and looked at other toys for 52% (SD ±11%) of the time a toy was in-hand, on average. A mixed-design ANOVA with proportion of frames in visual-manual behavior category (look at target, look at other toy) as a within-subjects factor and group (ASD, TD) as a between-subjects factor revealed a main effect of visual-manual behavior category (F(1, 27) = 13.29, p<0.001). Post-hoc Tukey tests revealed that a greater proportion of time was spent looking to other than target toys (target: 0.41 (0.12); other: 0.51 (0.10); t(56) = − 3.73, p<0.01, d = 0.98). However, there was not a significant interaction between visual-manual behavior category and group (F(1, 27) = 0.00, p = 1.00). Post-hoc Tukey tests revealed that there was no difference between groups in the proportion of time spent looking at the target (TD: 41% (12%); t(27) = -0.10, p = 0.92, d = -0.04) or the proportion of time spent looking at other toys (TD: 51% (8%); t(27) = 0.29, p = 0.77, d = 0.11) (See Fig. 4A,B). This suggests that children with and without ASD extend their visual attention beyond the toys that are the direct target of manual actions at similar frequencies.

#### Duration of looks during touch

We next aimed to determine if the duration of visual attention to target or other toys during in-hand events differed from each other or differed across groups. For each participant, we calculated the median duration of looks that overlapped with a manual action and were either directed to the target or to another toy. The average median duration of looks to target toys was 2.07s (SD±1.46s) for children with ASD, and the median look duration to other toys was 0.72s (SD ±0.11). For TD children, the average median duration of looks to target toys was 1.76s (SD±0.81s) and the median look duration to other toys was 0.76s (SD±0.31s). A mixed-design ANOVA with durations of look in visual-manual behavior category (look at target toy, look at other toy) as a within-subjects factor and group (ASD, TD) as a between-subjects factor revealed a main effect of visual-manual behavior category (F(1, 27) = 31.98, p<0.001). Post-hoc Tukey tests revealed that the duration of looks to target were longer than the duration of looks to other toys (target: 2.09s (1.28); other: 0.74 (0.22); t(56) = 5.63, p<0.01, d = 1.45). However, there was not a significant interaction between visual-manual behavior category and group (F(1, 2) = 0.69, p = 0.41). Post-hoc Tukey tests revealed that there was no difference between groups in the duration of looks to target (TD: 1.76s (0.81); t(27) = 0.71, p = 0.49, d = 0.26) or the duration of looks to other toys (TD: 0.76s (0.31); t(27) = -0.43, p = 0.67; d = 0.16) (See Fig. 4C,D). This suggests that for both groups, the greater proportion of time spent attending to other toys is comprised of many short looks whereas when looks are generated to toys being touched, these looks are longer in duration.

#### Looking behaviors at the moments before/after manual actions

**Figure 5:**
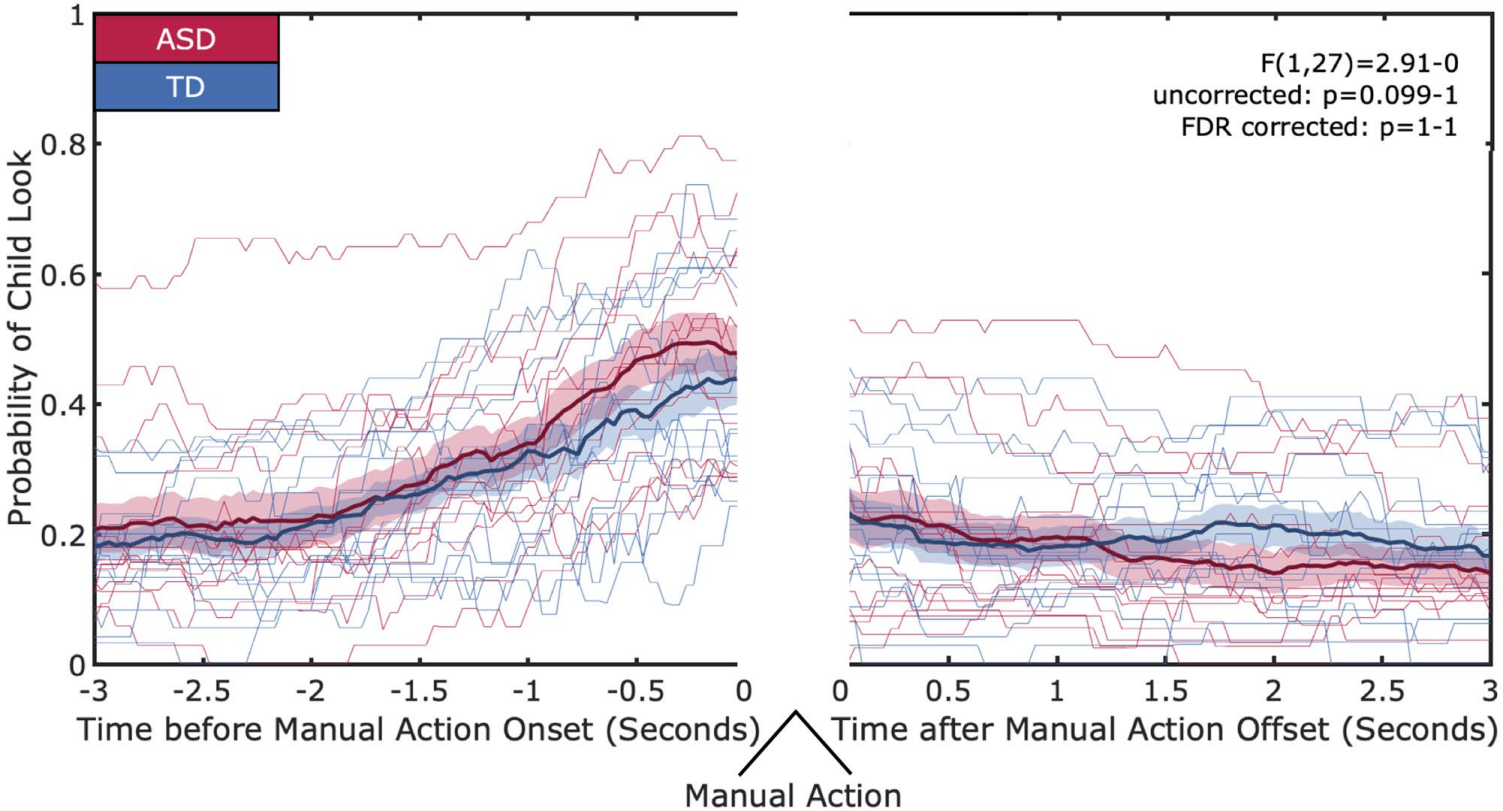
Hand-Eye Coordination. Each individual is represented by a thin line. The bold lines represent the group means, and the shaded region represents the standard error. The x-axis shows 3s before the onset of a manual action and 3s after the offset of a manual action. The y-axis shows the probability that the children are looking at the in-hand toy. There are no differences between groups in the likelihood of looking to the in-hand toy in the 3s before and after the manual action.

In an effort to understand the dynamic processes of the coordination of looks and manual actions, we zoomed into the moments before and after a toy was manually acted on and measured how the child’s looking behavior led to and followed the manual action. The present analysis is based on one used in psycho-linguistic studies to capture temporal profiles across a related class of events^38^. We measured frame-by-frame whether the child was looking at the target toy for 3s before an action onset and for 3s after an action offset, and then aggregated looking data across all instances of manual action. This resulted in a trajectory of the likelihood that the child was looking at the in-hand toy before and after the onset of manual action, which enables us to discern potentially important moments within a trajectory. The trajectory of the probability that the child was looking at the to-be-in-hand object shows a clear and dramatic increase as a function of temporal proximity to the child’s first contact, with the likelihood of visual attention to the target toy peaking around 0.5-0s before the onset of the manual action. In contrast, visual attention quickly switched away from the used-to-be-in-hand object after the termination of a manual action. To compare the trajectories extracted from children with ASD and TD children, we ran a generalized linear mixed effects model on each frame of the data predicting the number of actions where the child was looking at the toy with participant group as a fixed effect (comparing both groups to the mean) with random intercepts for each participant. Because the data are the number of touches where the participant was looking at the toy divided by the total number of touches, we used a binomial distribution in this analysis where the binomial size for each participant was the total number of touches. We found no difference in the likelihood that children looked to touched toys (F(1,27) = 2.91-0, uncorrected p = 0.10–1; FDR corrected all p = 1) (See Fig. 5). This suggests that dynamics of visual attention and motor action are similar between groups. For all children, visual attention tends to precede manual action. Further, manual action tends to persist beyond the termination of visual engagement in many cases.

Overall, we saw no differences between groups in the proportion of time attending to the in-hand toy compared to other toys. Looks generated to in-hand toys were longer overall, while looks to toys that were not currently in-hand were shorter. Visual attention preceding, during, and after a manual action were similar between children with ASD and their TD peers.

## Discussion

The current study employed a head-mounted eye tracking method to quantify how children with and without ASD distributed their visual attention and manual action over toys, and how attention and action were coordinated during play. Our first aim was to quantify how children with ASD distribute and coordinate their visual attention and manual action during play. Our second aim was to determine if previous findings reporting atypical visual attention could be generalized to a more naturalistic context. Our results suggest that young children with ASD are able and willing to explore toys during play with a caregiver, a result that is not consistent with past work showing a tendency for children with ASD to explore fewer objects for longer periods of time^14,15,18,25,26^. Over the course of 3 minutes of exploratory play, we saw no differences between children with ASD and their TD peers in their visual or manual exploration. They looked at and touched similar numbers of toys, they displayed a similar rate of sampling new toys from the environment, and they distributed their attention and actions across the toys in a similar way. Additionally, there were no differences in the influence of manual action on visual attention between groups. Children in both groups generated longer looks to toys being touched and shorter looks to toys not being touched, suggesting that children visually sample other toys in the environment while engaging with a toy in their hand. Further, children with ASD were not more likely to fixate on objects that were being touched. This does not coincide with past literature suggesting that children with ASD closely fixate on a single object for an extended time^14,21,22,39^. Below, we examine how space, time, and task are factors that may have contributed to the observed typical levels of exploration during dyadic play. Implications for intervention and research contexts and for developmental outcomes are discussed.

First, our study allowed children to play in an embodied context wherein they were able to move around the play space, manipulate objects, and view them from multiple angles. This stands in contrast to the passive viewing in screen-based eye tracking studies and physical limitations in play studies where the child is seated at a table. The participants in our study were able to employ manual action to promote their visual attention to toys, as well as move freely to explore their environments. Our results are consistent with some other studies that have found no differences between groups in the way that children explore objects when they are playing in more natural contexts, such as the child’s home, where the children are similarly able to move about the space^40–42^. Motor actions are executed on longer timescales than the eye movements that support visual attention. It is possible that visual attention in an unconstrained, embodied context may not reflect the abnormalities in visual attention that are observed at short timescales in constrained environments and tasks.

Second, the time-course of exploring toys during our study was self-paced as part of a free-flowing interaction. Children were able to move about their environment and actively choose toys for themselves from a large play set. This allowed the children to interact with toys that they found interesting and to choose to interact with a new toy at any moment. The different toy options and the ability to explore freely differs from the visual component of screen-based eye tracking studies where stimuli content and timing is pre-determined and from play studies that use just a few toys. Further, the set of toys with which the children and their parents played was composed of specific exemplars of toys that the children had not previously seen. This provides a situation in which it is adaptive to explore many of the toys before deciding what toy with which to play. Little is known about how restricted interests and behaviors develop, but it is possible that our study was not able to capture the onset of these behaviors.

Third, the current study set up a naturalistic play room-like setting for children to play with their caregiver with only the instructions to play together like they would at home. The results reported in this paper can be viewed as a joint product of parent-child interaction. Play is dynamic, such that the parent’s response to the child’s action becomes a new stimulus for the child to respond to, and so on^43,44^. The child’s actions can therefore be considered partially reflecting their own interest in toys and partially their response to the actions of the parent during play. The behaviors of the parent, while not directly explored in this analysis but an important topic for future work, may be effectively scaffolding seemingly typical levels of object exploration during toy play in children with ASD as compared to their TD peers. Parents also respond to their child’s actions and a typical level of exploration during play may suggest that children with ASD are able to give their parents equal opportunity to respond and continue the interaction. For example, when children play with toys, they often coordinate their gaze with toys that are in their hands^45,46^. This creates a clear opportunity for parents to join the child in looking at that object^45,46^, which has been shown to support the child’s attention to that toy^47,48^. Further, parents and children develop interaction styles together over time^12,13^, and it is likely that parents of children with ASD have learned some strategies for supporting their child during dyadic play.

Our observation of typical levels of object exploration during dyadic play holds many clinical and research implications. Context is extremely important to the study of behavior because different contexts elicit different kinds of behavior^16,41^. It would be important to directly compare object exploration during naturalistic free-play to behaviors during more constrained play-based interviews like the ADOS or to visual exploration behaviors in screen-based eye tracking. Such studies would help to elucidate how mechanisms underlying visual attention and exploration may be deferentially contributing to different task demands and contexts. Studying children in their natural environment during familiar activities such as play may allow us to capture both typical behaviors that provide important information regarding preserved or intact abilities in children with ASD and atypical behaviors that may create a developmental cascade of atypical cognitive and social abilities. Understanding both typical and atypical behaviors can be used to inform and refocus intervention efforts. Further, interventions that give the child more freedom to choose play spaces and play objects may be more rewarding to children with ASD and therefore support the learning of more typical behaviors. Our study introduced children to many toys in a short amount of time, and this could be leveraged in play-based interventions by introducing new toy sets frequently to help children practice and maintain adaptive exploration behaviors during play. For example, JASPER, a floor-play intervention that utilizes different sets of toys to facilitate child engagement, has been successful in improving play and social outcomes in children with ASD^49–55^. Additionally, our experimental context included the parent. While the behaviors that we observed appear typical, they may have developed through different dyadic pathways than the behaviors of typically developing children. Studying children in environments that more closely match their natural and social environments can help to quantify the child’s interactions with different affordances offered by the environment and the role of the parent in scaffolding these interactions. Studying children at play without a caregiver may also be advantageous to understanding the role of the parent in supporting toy play.

Our results may have implications for understanding how children with ASD experience and learn about the world. Exploration is linked to developmental outcomes—more typical object play is linked to better cognitive and language abilities in both groups^4,8–11,56,57^. Higher levels of object play during infancy were significantly positively correlated with later functional language skills in children with ASD^57^. Object exploration around two years of age predicted language abilities in children with and without ASD at around four years of age^56^. However, it is possible that both groups are able to gain some level of similar play input, but the processing of that input is different. By continuing to capture the dynamics of child play in a naturalistic setting, we may better be able to elucidate the mechanisms that might be underlying the onset of atypical attention in children with ASD despite an intact ability to explore toys in a typical way during early exploration. Specifically, research that incorporated the child’s favorite toy from home into the play set or studied the children for longer periods of time may be important to understanding perseverative attention or typical object exploration. Additionally, we employ a definition of manual action that makes the fewest assumptions as possible about the child’s goal in touching a toy. Instead, we treat hand contact with a toy as a feature of play that may direct a child’s attention or may reflect their interest in that toy. The current paper did not code for specific types of play or actions. Future planned analyses will aim to understand the different types of play that children engage in, including combinatorial play where two objects are played with at the same time. These future analyses, combined with those of the current paper, will allow us to further understand the types of actions that children use to guide and maintain their own attention. Further, we recognize that ASD is a heterogeneous disorder. We observed individual differences in all reported measures for children with and without ASD. One limitation to the use of head-mounted eye trackers is participant compliance with or tolerance of the procedures. It is possible that the children who were less tolerant of the eye tracker may exhibit different characteristics than those who were more tolerant. However, this did not seem to be the case, as the only difference observed between groups was that successfully tracked children had *higher* levels of restricted and repetitive behaviors. Thus, we believe that the sample of children with ASD who provided usable data is a representative sample of the ASD population and not biased toward a less severely affected subgroup. Relatedly, high levels of attrition contributed to the relatively small sample size in the present study, thus reducing statistical power. Thus, while we did not find evidence of differences between groups, it is possible that a larger sample would allow us to identify more subtle differences between ASD and TD groups. With improvements in data acquisition procedures, we expect that future studies using this methodology will be able to include increasingly larger numbers of participants. Future research with larger samples should examine whether individual differences in exploratory play within children with ASD are meaningful for predicting language, cognitive, and social developmental outcomes. Additionally, future research should aim to determine if individual differences across groups are related to outcomes in similar or different ways. This research will further help to elucidate the dichotomy between input and processing between groups.

In summary, the goal of the current study was to use head-mounted eye tracking to measure the dynamics of exploratory toy play in children with and without ASD. We found no differences between groups in their visual attention to toys, manual actions on toys, and visual-manual coordination. The current study is the first to our knowledge to use head-mounted eye tracking in dynamic contexts to study the behavior of children with ASD. Our findings suggest that studies of children in natural and embodied contexts may reveal disparate results from more constrained laboratory or clinical tasks. Our results further suggest that playing with a parent and with previously unplayed toys may facilitate typical visual and manual exploration behaviors. Future studies will aim to quantify the differences between screen-based and naturalistic exploration, the role of the parent in scaffolding play, and the role of new toy sets in supporting exploration as these may have impacts on the development of clinical interventions.

## Methods

### Participants

Fourteen children with ASD and 15 TD children contributed head-mounted eye-tracking data for the current study. Five children with ASD and 5 TD children were excluded from analyses for having insufficient data (defined as having less than 3 min of usable data). Twenty-eight children with ASD and 2 TD children were less tolerant of either wearing the eye tracker or allowing research personnel to make necessary adjustments. Subsequent protocol refinements (e.g., better distractors) and modifications to the equipment (e.g., better articulating arms holding the cameras, multiple cap sizes) resulted in improved success rates over the course of the project. Two children with ASD wore the eye tracker but the data was unusable. Unusable data were data where the child frequently bumped the eye tracker or had frequent depth shifts (i.e., moving from sitting to standing). These actions change the relationship between the eye movements and the images of the scene in front of the child. If segments of data were too short, the calibration of the eye image was unable to be completed. Eye tracking calibration is detailed in the Data Processing section below. An additional 4 ASD participants had equipment malfunctions and one additional TD child was excluded for vision concerns. The study aims were explained to all parents before participating and all parents provided informed consent. Parental consent was also obtained for the use of images collected during the study in scientific papers and presentations. Children with ASD who successfully wore the eye trackers were not significantly different in age, sex, or clinical presentation from those who did not (see Supplemental Table S1). Data collection for children with ASD was conducted at Cincinnati Children’s Hospital, and data collection for TD children was conducted at Indiana University, Bloomington. The two locations used the same testing equipment, stimuli, procedures, and personnel. ASD diagnosis was made by a trained clinician at Cincinnati Children’s Hospital according to the DSM-V criteria. The study was approved by the Indiana University Institutional Review Board and was conducted in accordance with all relevant guidelines and standards.

Table 1 provides characterization information for the ASD and the TD children included in the analyses. The ASD children were 37.23 months old on average (SD± 6.95) and the TD children were 37.03 months old (7.84). The two groups were not different on age, t(27) = 0.07, p = 0.95, d = 0.02. A chi-square test was performed to examine the relation between sex and group. The relationship between these variables was insignificant $\chi ^2$(N = 29) = 1.77, p = 0.18. The Autism Diagnostic Observation Scale-2 (ADOS) and Mullen Scales of Early Learning (Mullen) language subscales were administered to the ASD participants by trained, reliable personnel. If the child had previously completed the ADOS or a Mullen at Cincinnati Children’s Hospital within two years, those scores were used for the current study. Mullen Developmental Quotient (DQ) scores were computed as a normalized score of the child’s cognitive ability in both the receptive and expressive language domains. The DQ is computed by dividing the age equivalency by the child’s actual age at the Mullen and multiplying by 100. Therefore, a DQ of 100 would indicate that a child is performing at the level expected for their age. A DQ below 100 indicates deficits in language abilities. Of note, the children with ASD had both expressive and receptive language deficits as demonstrated by the averages and range (expressive range 13.0–84.9, receptive range 7.7–83.2) falling well below 100.

**Table 1:** Participant Characterization.

	ASD (n = 14)	TD (n = 15)
Age (months)	37.23 (SD ± 6.95)	37.03 (7.84)
Sex (M/F)	8/6	12/3
ADOS Social Affect	16.86 (5.11)	–
ADOS Restricted and Repetitive Behaviors	5.86 (3.53)	–
Mullen Expressive Language Developmental Quotient	48.97 (17.85)	–
Mullen Receptive Language Developmental Quotient	45.34 (16.46)	–

### Stimuli and procedure

Parents and their children were invited to a room decorated to resemble a toy room at home. A plush carpet was placed at the center of the room to serve as a play area. Dyads were asked to sit around the play area with their preferred seating arrangement. Many of them chose to sit across from each other with roughly a 90° angle and some other children preferred to sit on their parent’s lap. Once dyads were settled, we set up each dyad member with a head-mounted eye tracker (Positive Science, LLC). The eye trackers include an infrared camera that points to the participant’s right eye to capture movements of the eye and a small camera located on the participant’s forehead that captures the scene in front of him or her. Both cameras sampled data at 33 Hz. The eye tracker set-up is described in detail below. After eye tracker setup, the dyads were given a set of 24 everyday toys that were spread on the carpet. Parents were instructed to play with their child as they would at home with no explicit instruction to play with the toys.

The key step in our experimental procedure was equipping both the parent and the child with a head-mounted eye tracker. This step was accomplished by a primary experimenter and a second experimenter working together. First, the second experimenter and the parent engaged the toddler with an enticing toy with buttons to push that make animals pop up. The child’s head gear was placed by the primary experimenter while the child was engaged with the toy. This was done in one movement and care was taken by the experimenter to ensure that the child remained engaged with the toy and that the child’s hands didn’t go to the head gear. The first experimenter then adjusted the scene camera (visible in Fig. 1A, centered on the forehead between the eyes) to ensure that the camera angle was centered on the child’s actions. The next step was to adjust the infrared eye camera (visible in Fig. 1A, facing the right eye) to capture the child’s eye image. It is critical to position the eye camera to capture a clean image of the eye because the eye tracking calibration software relies on detecting the pupil and corneal reflection in order to project where participants look in their first-person view. Next, the child’s attention is systematically directed to different toys in the play area using a laser pointer as a starting point for off-line eye tracking calibration in later data pre-processing. During the experiment, one experimenter monitored the eye images on LCD monitors, if the eye tracker was touched or moved causing low quality in eye images, the experimenter entered the room and adjusted the eye tracker, and then the dyad resumed the study. The eye tracking apparatus does not occlude the view of the eyes, from either the child or the parent first-person perspectives. Finally, a previous study found that TD children who were not wearing the head-mounted eye trackers did not look to faces any differently than TD children wearing eye trackers during dyadic play^45^.

Following eye tracking data collection, experimenters performed a calibration procedure by which the participant’s eye movements were mapped onto the location in the scene camera where they were looking for each frame of the video. The first step of calibration was to identify segments of the data where the eye tracker was positioned on the eye in the same way and where the child was at the same depth from the ground. For example, a bump of the eye tracker will change the relationship between the eye movements and the x- and y-coordinates on the scene camera view. Additionally, the child standing or sitting changes the visual angle and requires a new calibration set to ensure calibration accuracy. Next, frames of the videos in which the participant picked up an object or moved their eyes to track an object were identified and indicated in the calibration software. These points allowed the software to map the location of the pupil to x- and y-coordinates of the scene camera. These points were distributed throughout session time and throughout the space captured by the scene camera and the calibration software interpolated the point of gaze in all additional frames. A purple crosshair represented the location of the participant’s eye gaze at each calibration point and in the frames in between (Fig. 1B). For each calibration, following current best practices in research with head-mounted eye tracking^58,59^, we validated calibration and re-calibrated using different calibration points if so indicated. Once a quality calibration was achieved, operationalized as the indicated calibration points having an intra-calibration correlation of 0.95 or greater, region-of-interest coding could be completed (see below).

### Data processing

Gaze region-of-interest (ROI) coding was done by highly trained human coders. The continuous gaze stream was segmented into individual looks based on eye movement velocity using a custom, in-house program. The eye gaze image was superimposed into the top right corner of the calibrated scene view, allowing coders to use both eye gaze information and the calibrated cross-hair for all subsequent coding. Coders examined the cross-hair and identified the target of each individual look. Targets were annotated when the cross-hair fell on a pixel identified as either one of the 24 toys or the social partner’s face.

Manual contact with an object was coded on a frame-by-frame basis using the child’s scene camera and other third-person camera views (shown in Fig. 1A). A custom program was used that allowed each coder to see the first-person and third-person views simultaneously, allowing for more accurate object identification. Contact with an object was coded each time the participant’s hand came in contact with one of the 24 toys. Two data streams were coded for each dyad member: one for the right hand and one for the left hand. When analyzing manual action data, manual actions on objects that used both hands at the same time were merged. A second coder independently coded a randomly selected subset of 5 participants and the inter-coder reliability ranged from 93% to 99% (Cohen’s kappa = 0.96).

### Data analysis

Data from 14 children with ASD and 15 TD children were included. In order to match the amount of data across participants, the first 3 min of usable data from each session were considered in the analyses. Unusable moments of data collection, such as any moments where the experimenter entered the interaction to adjust the eye tracker, were extracted from the data stream. Therefore, total exposure time to toys may be greater than the 3 min of usable data. The groups did not differ in the total exposure time to toys to achieve 3 min of usable data (ASD: 4.20 min (SD ± 1.42); TD: 4.45 min (1.79); t(26) = − 0.4, p = 0.69, d = 0.15). We ran generalized linear models on all outcome variables with toy exposure as a co-variate and found the effect of toy exposure on analyses was non-significant, with all p $\ge$ 0.496. Therefore, the reported analyses do not include toy exposure as a co-variate. We used an alpha level of .05 for all statistical tests.

## Supplementary Information

Supplementary Information.
