# Supplementary material for: Using head-mounted eye tracking to examine visual and manual exploration during naturalistic toy play in children with and without autism spectrum disorder
Source: Sci Rep. 2021 Feb 11;11:3578. doi: 10.1038/s41598-021-81102-0 (PMC7878779; doi:10.1038/s41598-021-81102-0)
Supplement: Supplementary file 1 — Supplementary Information. [file 41598_2021_81102_MOESM1_ESM.pdf]

Using Head-Mounted Eye Tracking to Examine Visual and Manual Exploration during Naturalistic Toy Play in Children with and without Autism Spectrum Disorder

Julia R. Yurkovic[1*], Grace Lisandrelli[1], Rebecca C. Shaffer[2,4], Kelli C. Dominick[3,4], Ernest V. Pedapati[3,4], Craig A. Erickson[3,4], Daniel P. Kennedy[1+*], and Chen Yu[1,5+*]

[1]Indiana University, Department of Psychological and Brain Sciences, Bloomington, IN, 47401, USA
[2]Cincinnati Children's Hospital, Department of Pediatrics, Cincinnati, OH 45229, USA
[3]Cincinnati Children's Hospital, Department of Psychiatry and Behavioral Neuroscience, Cincinnati, OH 45229, USA
[4]University of Cincinnati, School of Medicine, Cincinnati, OH 45229, USA
[5]University of Texas at Austin, Department of Psychological and Brain Sciences, Austin, Texas 78712, USA
[+]these authors contributed equally to this work
[*]Corresponding authors: jyurkovi@iu.edu, dpk@iu.edu, chen.yu@austin.utexas.edu

Supplementary Information

Table 1 provides characterization information for the ASD participants that were successful in wearing the eye-tracker compared to those who were unsuccessful. For the purpose of the current analysis, successful eye tracking was defined as wearing the eye tracker for the time it took to collect 3 minutes of usable data and therefore included only the 14 participants included in the main analyses. Unsuccessful participants were 28 participants who were unable to tolerate the eye tracker. The additional seven children who wore the eye tracker but contributed unusable or insufficient data were excluded from the current analysis, as well as the four children who were not given the opportunity to contribute data due to equipment malfunctions.

ASD Participants	Successful Eye Tracking (n=14)	Unsuccessful Eye Tracking (n=28)	*p*-Value	Cohen's d
Age (months)	37.23 (SD ± 6.95)	37.42 (8.25)	0.45	0.25
Sex (M/F)	9/5	21/7	0.48	—
ADOS Social Affect	16.86 (5.11)	16.46 (2.98)	0.75	0.10
ADOS Restricted and Repetitive Behaviors	5.86 (3.53)	3.96 (1.69)	0.02	0.77
Mullen Expressive Language Developmental Quotient	48.97 (17.85)	39.41 (17.79)	0.10	0.54
Mullen Receptive Language Developmental Quotient	45.34 (16.46)	36.67 (18.08)	0.13	0.49

Supplementary Table S 1. Characterization of ASD Participants with and without Successful Eye Tracking

Overall, there were no remarkable differences between participants who successfully wore the eye tracker compared to those who did not. The subgroups differed only on the ADOS Restricted and Repetitive Behaviors subscale, with successful participants demonstrating more restricted and repetitive behaviors overall than their unsuccessful peers. The subset of participants who contributed sufficient data are likely a representative subgroup of the larger sample of participants with ASD.
